# A functional haplotype of *NFKB1* influence susceptibility to oral cancer: a population‐based and in vitro study

**DOI:** 10.1002/cam4.1453

**Published:** 2018-04-10

**Authors:** Fa Chen, Fengqiong Liu, Lingjun Yan, Lisong Lin, Yu Qiu, Jing Wang, Junfeng Wu, Xiaodan Bao, Zhijian Hu, Lin Cai, Baochang He

**Affiliations:** ^1^ Department of Epidemiology and Health Statistics Fujian Provincial Key Laboratory of Environment Factors and Cancer School of Public Health Fujian Medical University Fuzhou China; ^2^ Key Laboratory of Ministry of Education for Gastrointestinal Cancer Fujian Medical University Fuzhou China; ^3^ Fujian Key Laboratory of Tumor Microbiology Fujian Medical University Fuzhou China; ^4^ Department of Oral and Maxillofacial Surgery The First Affiliated Hospital of Fujian Medical University Fuzhou China; ^5^ Laboratory Center The Major Subject of Environment and Health of Fujian Key Universities School of Public Health Fujian Medical University Fuzhou China

**Keywords:** biological mechanism, haplotype, *NFKB1*, *NFKBIA*, oral cancer

## Abstract

Genetic variations of NF‐κB and its inhibitor IκB genes and their biological mechanism in oral cancer were not well recognized. The purpose of this study was to evaluate the associations of polymorphisms in *NFKB1* and *NFKBIA* with oral cancer susceptibility, and further explore their potential mechanism in vitro. First, the polymorphisms of *NFKB1* and *NFKBIA* were genotyped through iPLEX Sequenom MassARRAY platform in a case–control study with 425 oral cancer patients and 485 healthy controls. Then, the function was explored by a luciferase reporter assay and an electrophoretic mobility shift assay (EMSA) in human tongue squamous cell carcinoma cell lines. The results indicated that *NFKB1* rs28362491 Del/Del and rs72696119 G/G genotypes were associated with the risk of oral cancer, with a strong linkage disequilibrium (*D*′ = 0.991, *r* ^2^ = 0.971). Moreover, DG haplotype of *NFKB1* also showed a significant increased risk (OR = 1.25, 95% CI: 1.02–1.53, *P* = 0.030). Dual‐luciferase reporter assays further revealed that the plasmids with DG or IG or DC haplotype transfected with Tca‐8113 cells or CAL‐27 cells had a lower luciferase expression than that with IC haplotype. EMSA demonstrated that 4‐bp ATTG deletion in the promoter of *NFKB1* abolished the binding site of transcription factor. Our preliminary findings suggest that the haplotype of rs28362491 and rs72696119 in *NFKB1* could act as a novel genetic marker to predict oral cancer risk in the southeast of China, but much more extensive researches still need to be conducted.

## Introduction

Oral cancer, the most common cancer in head and neck, is a growing public health problem in many parts of the world especially for developing countries 1, 2. Our previous epidemiological studies have suggested that environmental factors such as cigarette smoking, alcohol drinking, tea consumption, dietary factors, oral hygiene, and HPV infection were associated with the risk of oral cancer 3, 4, 5, 6, 7. However, despite exposure to the same environmental factors, only a few individuals develop oral cancer, indicating that genetic factors also play a critical role in oral cancer.

Single‐nucleotide polymorphism (SNP) is one of the most common types of human genetic variation. Various SNPs were reported to be correlated with the susceptibility of oral cancer 8, 9. Recently, the polymorphisms of the NF‐κB signaling pathway have been widely studied due to the close relationship between inflammation and cancer 10, 11. NF‐κB is a central regulator of inflammation and cancer development, and its family consists of five members: NF‐κB1 (p50), NF‐κB2 (p52), Rel A (p65), Rel B and c‐Rel. The heterodimer of the p50/p65 is the most common form of NF‐κB, which is encoded by the *NFKB1* and *RelA* genes, respectively 12. In resting cells, NF‐κB binds to its inhibitors IκBs (the most common protein is IκBα, encoded by *NFKBIA*) and remains inactive in cytoplasm. After IκBs are phosphorylated and degraded by various stimuli, NF‐κB is translocated into the nucleus and activated the transcription of target genes 13, 14.

Previous studies have demonstrated that several genetic variations of *NFKB1* and *NFKBIA* were associated with cancer risk such as esophageal 15, gastric 16, and colorectal cancer 17. However, to date, few studies have reported the link between genetic polymorphisms and oral cancer risk 18. Moreover, limited experimental studies have been conducted to explore the biological mechanism of *NFKB1* polymorphisms on susceptibility of oral cancer.

Therefore, this study aims to examine the association of *NFKB1* and *NFKBIA* genes polymorphisms with the susceptibility to oral cancer in southeast China, and further explore their biological mechanism on oral cancer in vitro.

## Materials and Methods

### Study participants

From January 2009 and May 2016, a case–control study was conducted in Fujian province, China. As previously described 9, 427 oral cancer patients were recruited from the First Affiliated Hospital of Fujian Medical University. The inclusion criteria of the patients were as follows: (1) all cases were newly diagnosed with histologically confirmed; (2) all cases live in Fujian Province and aged 20–80 years. Patients with second primary, recurrent or metastasized cancer, and previous radiotherapy or chemotherapy were excluded.

A total of 488 healthy controls were randomly selected from medical examination center in the same hospital and were frequency matched for age and gender to the cases. Those with history of cancer were excluded. Written informed consent was obtained from all participants. This study was performed in line with the ethical standards described in the 1964 Declaration of Helsinki and approved by the Institutional Review Board of Fujian Medical University (Fuzhou, China).

The sample size was calculated by PASS software (version 11). Based on previous studies, we set *α* as 0.05, β as 0.90, *P* as 0.3, and OR as 1.55. The required sample size was at least 400. Therefore, the sample size in this study satisfied the research needs.

### Data and sample collections

Epidemiological data were collected by well‐trained interviewers through face‐to‐face interview using a standardized questionnaire. The questionnaire included demographic characteristics, smoking, drinking, oral hygiene status, residential history, and family history of cancer. Oral hygiene score was calculated based on related variables including tooth brushing, wearing dentures, the number of missing teeth, regular dental visits, and recurrent oral ulceration 6; 3–5 mL blood samples were collected from all enrolled subjects with EDTA‐containing vacuum tubes and stored at −80°C.

### SNPs selection and genotyping

Tagging SNPs in *NFKB1* and *NFKBIA* were selected from the National Center for Biotechnology Information (NCBI) database (http://www.ncbi.nlm.nih.gov/projects/SNP) according to the following conditions: (1) the minor allele frequency (MAF) for all tagging SNPs are more than 0.05 in Chinese Han population; (2) Hardy–Weinberg equilibrium *P*‐value analyzed by Haploview software also more than 0.05. Finally, we identified five tagging SNPs, rs28362491, rs72696119, rs696, rs2233406, and rs2273650, for further analysis. Genomic DNA of each subjects was extracted from whole‐blood samples using TIANamp Genomic DNA Kit (Tiangen Biotech, Beijing, China). Genotyping was performed by iPLEX Sequenom MassARRAY platform (Sequenom, San Diego, CA) according to the recommended protocols. Genotyping results were then automatically analyzed by the TYPER 4.0 software. For quality control, three no‐template controls were performed in each 384‐well plate. About 5% of the DNA samples were randomly selected to re‐genotype and the concordance rate reached 100%. The success genotyping rates of all SNPs were over 99%. As a result of genotyping failure of some DNA samples, a total of 425 cases and 485 controls were included for further analysis.

### Luciferase activity assay

About −800 to 60 region of the *NFKB1* promoter containing −94 Ins allele (rs28362491) and −449 C allele (rs72696119) were synthesized and constructed into the pGL3‐Basic reporter plasmid (Promega, Madison, WI) named PGL3‐NFKB1‐IC. Using site‐directed mutagenesis technique, PGL3‐NFKB1‐IC was then mutated to PGL3‐NFKB1‐DC, PGL3‐NFKB1‐IG, and PGL3‐NFKB1‐DG, respectively. Sequence analysis was performed to confirm these plasmids. Subsequently, human tongue squamous cell carcinoma cell lines Tca‐8113 (Procell, Wuhan, China) and CAL‐27 (Procell) in 12‐well plates were transfected with reporter plasmids (1.0 μg) and plasmid pRL‐TK (1.0 μg) (Promega) as an internal control containing renilla luciferase reporter gene. After 12 h, luciferase activities were detected using a dual‐luciferase assay kit (Beyotime, Beijing, China) following the manufacturer's instructions. Relative luciferase activity was calculated according to the RLU (relative light unit) of the firefly luciferase divided by the RLU of the renilla luciferase.

### Electrophoretic mobility shift assays

Biotin 3′‐end labeled and unlabeled oligonucleotides covering *NFKB1* SNP rs28362491 were synthesized (Sangon Biotech, Shanghai, China). The sequences for Ins allele and Del allele were 5′‐TCCCCGACCATTGATTGGGCCCGGC‐3′ and 5′‐ TCCCCGACCATTGGGCCCGGC‐3′, respectively. Nuclear extracts from Tca‐8113 cells were prepared using Nuclear and Cytoplasmic Protein Extraction Kit (KeyGen, Jiangsu, China). Then, EMSAs were performed following the Light Shift Chemiluminescent EMSA Kit (Thermo Pierce, Rockford, IL). The nuclear extracts (5 μg) and labeled probes (200 fmol) were incubated with a volume of 20 μl reaction including 2 μL of 10% binding buffer, 1 μL of 2.5% glycerol, 5 mmol/L MgCl_2_, 50 ng/μL poly (dI–dC), and 0.05% NP‐40 for 20 min at room temperature. For competition assay, unlabeled probes with 200‐fold molar excess were added in the binding reaction. Subsequently, nondenaturating 6% polyacrylamide gel electrophoresis in 0.5× TBE running buffer were performed and finally exposed with X‐ray film.

### Statistical analysis

The distributions of demographics and main lifestyle factors between cases and controls were compared by chi‐square test. Hardy–Weinberg equilibrium of selected SNPs among control subjects were assessed using a goodness‐of‐fit chi‐square test. Unconditional logistic regression models were used to calculate odds ratios (ORs) and their 95% confidence intervals (CIs) for evaluating the associations of SNPs with oral cancer risk. The frequencies of haplotypes were calculated, and the haplotype block was constructed using Haploview 4.2 19. The multiplicative interactions of SNPs with environmental factors were assessed by logistic regression analysis. Additive interactions were tested using the relative excess risk due to interaction (RERI), attributable proportion (AP), and synergy index (SI). The levels of luciferase activity between different groups were compared by one‐way ANOVA test. All analyses were carried out using R software (version 3.1.1). The level of statistical significance was set at *P* < 0.05.

## Results

The main characteristics of cases and controls are listed in Table 1. There were no significantly different distributions of age, gender, and marital status between cases and controls (*P* > 0.05). In regard to the major risk factors, oral cancer patients were probable to have tobacco smoking, alcohol drinking, and poor oral hygiene than controls (*P* < 0.05).

**Table 1:** Distribution of demographic characteristics and the main risk factors among cases and controls

Variable	Case (%) (*n* = 425)	Control (%) (*n* = 485)	*χ* ^2^	*P* value
Age (years)			8.49	0.075
<40	38 (8.94)	46 (9.48)		
40–	86 (20.24)	114 (23.51)		
50–	125 (29.41)	168 (34.64)		
60–	109 (25.65)	102 (21.03)		
≥70	67 (15.76)	55 (11.34)		
Gender			3.37	0.066
Male	269 (63.29)	278 (57.32)		
Female	156 (36.71)	207 (42.68)		
Education level			30.39	<0.001
Primary and below	173 (40.71)	170 (35.05)		
Middle school	210 (49.41)	200 (41.24)		
College and above	42 (9.88)	115 (23.71)		
Marital status			2.44	0.118
Married	383 (90.12)	451 (92.99)		
Others	42 (9.88)	34 (7.01)		
Residence			76.54	<0.001
Rural	235 (55.29)	130 (26.80)		
Urban	190 (44.71)	355 (73.20)		
Tobacco smoking			41.34	<0.001
No	212 (49.88)	343 (70.72)		
Yes	213 (50.12)	142 (29.28)		
Alcohol drinking			39.04	<0.001
No	258 (60.71)	386 (79.59)		
Yes	167 (39.29)	99 (20.41)		
Oral hygiene score			52.35	<0.001
0–3	146 (34.35)	283 (58.35)		
4–8	279 (65.65)	202 (41.65)		

The association between genotypes and oral cancer risk is shown in Table 2. The distributions of *NFKB1* and *NFKBIA* genotypes were in Hardy–Weinberg equilibrium (all *P* > 0.05). *NFKB1* rs28362491 Del/Del genotype was associated with a 1.57‐fold increased risk of oral cancer (adjusted OR = 1.57, 95% CI: 1.07–1.92, *P* = 0.030). Moreover, compared with Ins/Ins or Ins/Del carriers, subjects with Del/Del homozygous allele had a significantly higher risk of oral cancer. Additionally, an increased risk was also found for GG genotype of rs72696119, compared with CC genotype (adjusted OR = 1.67, 95% CI: 1.11–2.50, *P* = 0.014). However, *NFKBIA* rs696, rs2233406, and rs2273650 polymorphisms were not observed to be associated with oral cancer risk.

**Table 2:** *NFκB1* and *NFKBIA* polymorphisms and risk of oral cancer

Gene locus	Genotypes	Case (%) (*n* = 425)	Control (%) (*n* = 485)	Adjusted OR^a^ (95% CI)	*P* value	PAR%
rs28362491 (*P*_HWE_ = 0.577)						
Codominant model	Ins/Ins	124 (29.45)	163 (33.75)	1.00		
	Ins/Del	197 (46.80)	230 (47.62)	0.99 (0.71–1.38)	0.959	−0.48
	Del/Del	100 (23.75)	90 (18.63)	1.57 (1.04–2.37)	0.030	9.60
Dominant model	Ins/Del + Del/Del vs. Ins/Ins			1.14 (0.84–1.56)	0.406	8.49
Recessive model	Del/Del vs. Ins/Ins + Ins/Del			1.58 (1.10–2.26)	0.012	9.75
Allelic model^b^	Del vs. Ins			1.21 (1.01–1.46)	0.045	8.18
rs72696119 (*P*_HWE_ = 0.184)						
Codominant model	CC	125 (29.69)	169 (35.28)	1.00		
	CG	193 (45.84)	219 (45.72)	1.05 (0.75–1.46)	0.794	2.23
	GG	103 (24.47)	91 (19.00)	1.67 (1.11–2.50)	0.014	11.29
Dominant model	CG + GG vs. CC			1.21 (0.89–1.65)	0.226	11.97
Recessive model	GG vs. CC + CG			1.62 (1.14–2.32)	0.008	10.54
Allelic model^b^	G vs. C			1.25 (1.04–1.51)	0.019	9.47
rs696 (*P*_HWE_ = 0.943)						
Codominant model	CC	130 (30.81)	153 (31.68)	1.00		
	TC	216 (51.18)	237 (49.07)	1.03 (0.75–1.43)	0.841	1.45
	TT	76 (18.01)	93 (19.25)	0.85 (0.56–1.30)	0.464	−2.97
Dominant model	TC + TT vs. CC			0.98 (0.72–1.34)	0.909	−1.39
Recessive model	TT vs. CC + TC			0.84 (0.58–1.21)	0.348	−3.18
Allelic model^b^	T vs. C			0.99 (0.82–1.20)	0.936	−0.44
rs2233406 (*P*_HWE_ = 0.931)						
Codominant model	GG	308 (72.47)	365 (75.57)	1.00		
	GA	108 (25.41)	110 (22.77)	1.16 (0.83–1.63)	0.376	3.52
	AA	9 (2.12)	8 (1.66)	1.28 (0.43–3.79)	0.660	0.46
Dominant model	GA + AA vs. GG			1.18 (0.86–1.63)	0.344	4.21
Recessive model	AA vs. GG + GA			1.22 (0.43–3.45)	0.712	0.36
Allelic model^b^	A vs. G			1.16 (0.89–1.51)	0.274	2.10
rs2273650 (*P*_HWE_ = 0.672)						
Codominant model	CC	225 (53.19)	256 (53.11)	1.00		
	TC	167 (39.48)	188 (39.01)	0.96 (0.71–1.30)	0.792	−1.59
	TT	31 (7.33)	38 (7.88)	1.03 (0.59–1.80)	0.907	0.24
Dominant model	TC + TT vs. CC			0.97 (0.73–1.30)	0.846	−1.43
Recessive model	TT vs. CC + TC			1.05 (0.61–1.80)	0.855	0.39
Allelic model^b^	T vs. C			0.98 (0.80–1.21)	0.880	−0.55

a Adjusted for age, gender, education, ethnicity, marital status, residence, smoking, drinking, and oral hygiene.

b The results were crude OR (95% CI).

Then, when stratified by demographics and main risk factors, the increased risk associated with Del/Del or GG genotype tended to be more evident in smokers (Table 3). Moreover, a strong linkage was found between rs28362491 and rs72696119 (*D*′ = 0.991, *r* ^2^ = 0.971). Haplotype analyses of *NFKB1* gene demonstrated that IC was the most frequent haplotypes (52.64% for cases and 57.41% for controls), and DG haplotype carriers had a significantly increased risk of oral cancer than subjects with IC haplotype (adjusted OR = 1.25, 95% CI: 1.02–1.53, *P* = 0.030, Table 4).

**Table 3:** Stratified analysis for oral cancer risk associated with genotypes of rs28362491 and rs72696119

Variables	rs28362491 (DelDel vs. InsIns + InsDel)		rs72696119 (GG vs. CC + CG)	
	Adjusted OR (95% CI)^a^	*P* value	Adjusted OR (95% CI)^a^	*P* value
Age (years)				
<60	1.91 (1.18–3.10)	0.008	2.02 (1.26–3.26)	0.004
≥60	1.09 (0.61–1.95)	0.783	1.08 (0.60–1.94)	0.791
Gender				
Male	1.88 (1.15–3.07)	0.012	1.83 (1.12–2.98)	0.016
Female	1.19 (0.69–2.05)	0.528	1.33 (0.78–2.27)	0.301
Education level				
Primary school and below	1.12 (0.64–1.95)	0.701	1.07 (0.61–1.88)	0.811
Middle school	2.15 (1.25–3.71)	0.006	2.35 (1.37–4.04)	0.002
College and above	1.10 (0.35–3.41)	0.872	1.06 (0.34–3.30)	0.916
Residence				
Rural	1.36 (0.78–2.36)	0.275	1.33 (0.77–2.31)	0.307
Urban	1.74 (1.07–2.81)	0.025	1.83 (1.14–2.94)	0.013
Tobacco smoking				
No	1.27 (0.82–1.96)	0.291	1.37 (0.89–2.11)	0.148
Yes	2.00 (1.05–3.80)	0.034	1.93 (1.02–3.65)	0.044
Alcohol drinking				
No	1.45 (0.97–2.18)	0.069	1.42 (0.96–2.12)	0.081
Yes	1.68 (0.81–3.49)	0.167	1.49 (0.74–3.01)	0.265
Oral hygiene index				
0–3	1.95 (1.16–3.30)	0.012	1.96 (1.16–3.33)	0.012
4–8	1.25 (0.76–2.05)	0.383	1.30 (0.79–2.13)	0.296

a Adjusted for age, gender, education, ethnicity, marital status, residence, smoking, drinking, and oral hygiene.

**Table 4:** Association between *NFκB1* haplotypes and oral cancer risk

Haplotypes	Cases (%)	Controls (%)	Adjusted OR (95% CI)^a^	*P* value
rs28362491–rs72696119				
IC	439 (52.64)	550 (57.41)	1.00	
DG	391 (46.88)	399 (41.65)	1.25 (1.02–1.53)	0.030
IG/DC	4 (0.48)	9 (0.94)	0.67 (0.19–2.39)	0.542

a Adjusted for age, gender, education, ethnicity, marital status, residence, smoking, drinking, and oral hygiene.

Further, genetic risk score (GRS) was calculated by summing the number of risk alleles (Del or G) of rs28362491 and rs72696119. A significant tendency was observed for increased risk with the increasing number of GRS (*P* trend < 0.05, Table 5). Additionally, gene‐environmental interaction analysis revealed a multiplicative effect between GRS and smoking or drinking or oral hygiene, but not for additive effect (Table 6).

**Table 5:** Association between the genetic risk score of *NFκB1* and oral cancer risk

Variables	Cases (%)	Controls (%)	Adjusted OR (95% CI)^a^	*P* value
Genetic risk score				
0	123 (29.50)	162 (33.82)	1.00	
1	2 (0.48)	7 (1.46)	0.31 (0.05–1.96)	0.212
2	191 (45.80)	220 (45.93)	1.01 (0.71–1.40)	0.992
3	2 (0.48)	0 (0.00)	NA	NA
4	99 (23.74)	90 (18.79)	1.56 (1.03–2.36)	0.034

NA, data not available.

a Adjusted for age, gender, education, ethnicity, marital status, residence, smoking, drinking, and oral hygiene.

**Table 6:** Interactions of *NFκB1* polymorphisms with the main risk factors on oral cancer

Variables	GRS (0–2)	GRS (3–4)	OR_mutiplacative_ (95% CI)^a^	RERI (95% CI)^a^	AP (95% CI)^a^	S (95% CI)^a^
	Adjusted OR (95% CI)^a^	Adjusted OR (95% CI)^a^				
Tobacco smoking			2.40 (1.27–4.53)	1.46 (−1.51 to 4.43)	0.31 (−0.14 to 0.77)	1.67 (0.70–4.01)
No	1.00	1.56 (1.01–2.41)				
Yes	2.62 (1.66–4.14)	4.64 (2.29–9.39)				
Alcohol drinking			2.31 (1.14–4.67)	0.70 (−1.69 to 3.08)	0.21 (−0.38 to 0.80)	1.44 (0.47–4.36)
No	1.00	1.61 (1.07–2.43)				
Yes	1.99 (1.30–3.04)	3.30 (1.59–6.84)				
Oral hygiene score			1.70 (1.05–2.76)	−0.40 (−2.36 to 1.55)	−0.12 (−0.72 to 0.49)	0.86 (0.40–1.83)
0–3	1.00	2.10 (1.27–3.48)				
4–8	2.76 (1.91–3.97)	3.45 (1.99–5.98)				

a Adjusted for age, gender, education, ethnicity, marital status, residence, smoking, drinking, and oral hygiene.

**Figure 1:**
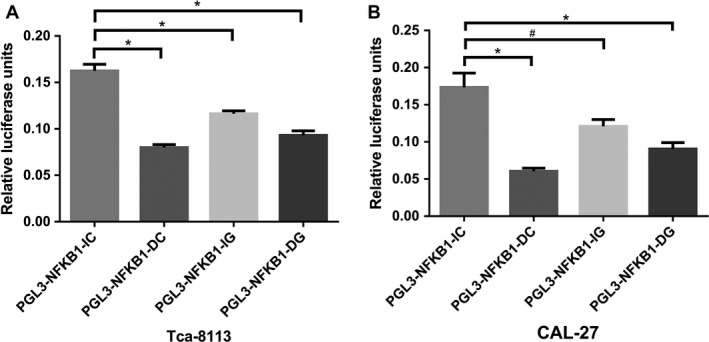
The relative activity of *NFKB1* promoter‐double luciferase reporter. A, in Tca‐8113 cell lines; B, in CAL‐27 cell lines. *P<0.001, ^#^ *P* = 0.007.

We further constructed *NFKB1* promoter luciferase reporter plasmids containing rs28362491 Ins/Del and rs72696119 C/G. Dual‐luciferase reporter assay showed that Tca‐8113 cells transfected with DG or IG or DC plasmids had lower luciferase activity than that of cells transfected with IC plasmid (*P* < 0.001, Fig. 1A). Similar results were observed in CAL‐27 cells, and all promoters with the three plasmids also showed lower activity than that of the IC plasmid (Fig. 1B).

**Figure 2:**
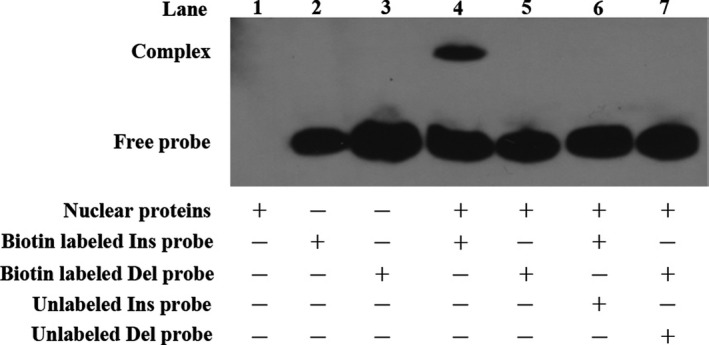
Electrophoretic mobility shift assays (EMSAs) for *NFKB1* rs28362491 polymorphism. Protein binding activities of the 6 *NFKB1* promoter mimic oligonucleotide probes. Lane 1: nuclear proteins derived from Tca‐8113 cell lines; Lane 2: Biotin‐labeled Ins allele probe; Lane 3: Biotin‐labeled Del allele probe; Lane 4: nuclear proteins + biotin‐labeled Ins allele probe; Lane 5: nuclear proteins + biotin‐labeled Del allele probe; Lane 6: nuclear proteins + biotin‐labeled Ins probe + unlabeled Ins probe; Lane 7: nuclear proteins + biotin‐labeled Del probe + unlabeled Del probe.

We subsequently performed electrophoretic mobility shift assays (EMSAs) to explore the influence of *NFKB1* rs28362491 polymorphism on nuclear proteins binding in Tca‐8113 cells. The differential patterns of nuclear proteins binding are presented in Figure 2. I allele probe had strong binding activity to nuclear protein of Tca‐8113 cells and formed a complex band (Fig. 2, line 4), but the mutant D allele probe showed no binding to nuclear protein (Fig. 2, line 5).

## Discussion

To our knowledge, this case–control study was the first to explore the associations and their potential mechanism of a functional haplotype in *NFKB1* (rs28362491 and rs72696119) with oral cancer susceptibility in a Chinese population. Our findings suggested that either rs28362491 Del/Del or rs72696119 GG polymorphisms was associated with an increased risk of oral cancer. Moreover, DG haplotype of *NFKB1* also showed a significant increased risk. In vitro, DG haplotype had the lower transcriptional activity in luciferase reporter systems. Additionally, deletion of *NFKB1* −94 ATTG led to the loss of binding to nuclear protein.

rs28362491 is a functional polymorphism in the promoter region of *NFKB1* gene. Our results observed a significant association between rs28362491 Del variation and the increased risk of oral cancer. In contrast, a recent study showed that Ins allele was related to oral squamous cell carcinoma risk 18. Gupta et al. 20 also found the same effect of Ins/Ins genotype on head and neck in an Indian population. This difference may be due to diverse genetic background and interactions with environmental factors such as betel nut chewing and smoking, which vary widely in different regions. Betel nut chewing has a very low prevalence in southeastern China, and smoking is the major risk factors for oral cancer in the area. Tobacco smoke could activate and up‐regulate the expression of NF‐κB in oral keratinocytes 21. Additionally, we speculated that the oral mucosa of Del/Del genotype carriers is more susceptible to tobacco carcinogens, but the specific mechanism needs to be clear in further study.

According to the results of EMSA and dual‐luciferase reporter assay, we inferred that the mechanism for rs28362491 polymorphism affects the susceptibility of oral cancer may be attributed to the Del allele of the polymorphism resulting in the abolition of the nuclear protein binding site leading to reducing promoter activity 22. Subsequently, the production of p50 is reduced, resulting in a corresponding decrease in p50/p50 homodimer, which has anti‐inflammatory properties and facilitates the expression of anti‐inflammatory cytokine 23. Conversely, p65/p50 heterodimer with pro‐inflammatory effect is relatively increased, which could stimulate the transcription of several pro‐inflammatory cytokines, such as TNFα, IL‐1, and IL‐12. Sustained activation of NF‐κB1 could induce a series of inflammatory responses 24, 25. Therefore, rs28362491 Del variation will be unfavorable for the anti‐inflammatory response and facilitates a higher pro‐inflammatory status, which may cause the occurrence and progression of oral cancer.

rs72696119 is located in the 5′‐UTR of *NFKB1* gene. In the present study, rs72696119 GG homozygote was found to be associated with oral cancer risk. Although there are no reports so far for the association of this polymorphism with oral cancer, previous studies indicated that GG genotype significantly increased the risk of gastric cancer and ulcerative colitis 26, 27. Additionally, we noticed a strong linkage disequilibrium between rs28362491 and rs72696119, which is consistent with a recent study 28. Moreover, DG haplotype of *NFKB1* had a 1.25‐fold risk of developing oral cancer compared with IC haplotype. We further explored the mechanism by which the DG haplotype was found to reduce the transcription activity of *NFKB1*, which may provide a possible explanation for rs72696119 polymorphism in the etiology of oral cancer.

However, there are several potential limitations in this study. First, we only focused on the variants of two classic genes (*NFKB1* and *NFKBIA*) in NF‐κB signaling pathway, and future studies should cover other polymorphisms and candidate genes in this pathway. Second, we only conducted a single‐center study in southeast of China, and the extrapolation of the results remains to be determined. Given the numbers in each group evaluated got quite small for some of the comparisons, this study is still relatively small and validation in larger sample studies with ethnic diversity is needed. Third, although the EMSA showed that 4‐bp ATTG deletion in the promoter of *NFKB1* abolished the binding site of transcription factor, further studies still need to determine the type of transcription factor.

In conclusion, this preliminary study demonstrates that the haplotype of rs28362491 and rs72696119 in *NFKB1* could act as novel genetic markers to predict the risk of oral cancer in the southeast of China. rs28362491 polymorphism may modulate oral cancer risk by changing the transcription factor binding pattern. Our study could provide an additional evidence for the function of *NFKB1* gene on oral cancer risk, and help understand the molecular mechanisms of oral cancer. Much additional and more extensive work are still warranted to perform.

## Conflict of Interest

None declared.
